# Digital droplet RT‐LAMP increases speed of SARS‐CoV‐2 viral RNA detection

**DOI:** 10.1002/SMMD.20240008

**Published:** 2024-06-05

**Authors:** Yuan Yuan, Perry Ellis, Ye Tao, Dimitri A. Bikos, Emma K. Loveday, Mallory M. Thomas, James N. Wilking, Connie B. Chang, Fangfu Ye, David A. Weitz

**Affiliations:** ^1^ Oujiang Laboratory (Zhejiang Lab for Regenerative Medicine, Vision and Brain Health) Wenzhou Institute University of Chinese Academy of Sciences Wenzhou Zhejiang China; ^2^ John A. Paulson School of Engineering and Applied Sciences Harvard University Cambridge Massachusetts USA; ^3^ Department of Chemical and Biological Engineering Montana State University Bozeman Montana USA; ^4^ Center for Biofilm Engineering Montana State University Bozeman Montana USA; ^5^ Department of Physiology and Biomedical Engineering Mayo Clinic Rochester Minnesota USA; ^6^ Beijing National Laboratory for Condensed Matter Physics Institute of Physics Chinese Academy of Sciences Beijing China; ^7^ Department of Physics Harvard University Cambridge Massachusetts USA; ^8^ Wyss Institute for Biologically Inspired Engineering Harvard University Boston Massachusetts USA

**Keywords:** ddRT‐LAMP, droplet microfluidics, droplet size, RNA rapid detection, SARS‐CoV‐2 virus

## Abstract

Nucleic acid amplification testing (NAAT) remains one of the most reliable methods for pathogen identification. However, conventional bulk NAATs may not be sufficiently fast or sensitive enough for the detection of clinically‐relevant pathogens in point‐of‐care testing. Here, we have developed a digital droplet RT‐LAMP (ddRT‐LAMP) assay that rapidly and quantitatively detects the SARS‐CoV‐2 viral E gene in microfluidic drops. Droplet partitioning using ddRT‐LAMP significantly accelerates detection times across a wide range of template concentrations compared to bulk RT‐LAMP assays. We discover that a reduction in droplet diameter decreases assay times up to a certain size, upon which surface adsorption of the RT‐LAMP polymerase reduces reaction efficiency. Optimization of drop size and polymerase concentration enables rapid, sensitive, and quantitative detection of the SARS‐CoV‐2 E gene in only 8 min. These results highlight the potential of ddRT‐LAMP assays as an excellent platform for quantitative point‐of‐care testing.

## Graphical abstract

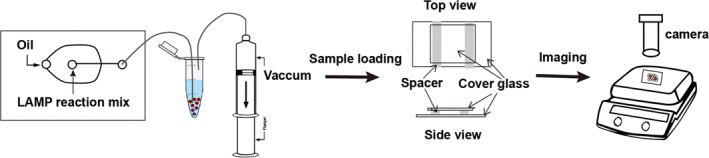

**Key points**

- This study presents the development of a digital droplet RT‐LAMP (ddRT‐LAMP) assay for the rapid and quantitative detection of the SARS‐CoV‐2 viral E gene.
- Decreasing droplet size reduced assay times up to a certain threshold beyond which surface adsorption of the RT‐LAMP polymerase hamper reaction efficiency.
- By optimizing drop size and polymerase concentration, our approach achieved swift, sensitive, and quantitative detection of the SARS‐CoV‐2 E gene in just 8 min.

## 1 INTRODUCTION

Nucleic acid amplification testing (NAAT) is widely regarded as the gold standard for detecting genetic material and is extensively used in clinical diagnostics.^1^, ^2^, ^3^, ^4^ During the COVID‐19 pandemic, laboratory‐based NAATs exhibited the highest sensitivity and specificity in detecting the SARS‐CoV‐2 virus compared to other diagnostic methods, such as antigen tests.^5^, ^6^ NAATs function by amplifying the target nucleic acids into detectable quantities. However, when target nucleic acids are present in low concentrations within a specimen, their amplification may require more time or even fail to yield a detectable signal, depending on the sensitivity of the instrumentation.^7^ For example, conventional bulk NAATs typically require assay times of 45 min to 1 h, or even longer, to detect clinically‐relevant viral loads of SARS‐CoV‐2.^8^ This makes them fundamentally unsuited for rapid point‐of‐care testing. Consequently, point‐of‐care testing during the COVID‐19 pandemic up to the present day has been dominated by rapid antigen tests, which provide results within 15 min.^5^, ^6^ However, these tests are much less sensitive and cannot quantify viral load compared to NAATs. An ideal solution for point‐of‐care diagnostics would combine the speed of a rapid antigen test with the sensitivity and quantification capabilities of a NAAT.

Reverse‐transcription Loop‐Mediated Isothermal Amplification (RT‐LAMP) is a rapid, isothermal method for RNA amplification that uses four to six primers to enhance assay specificity.^9^, ^10^, ^11^, ^12^, ^13^ Operating at a constant temperature between 60°C and 65°C, RT‐LAMP^11^, ^12^, ^13^ offers high specificity, relatively high sensitivity, lower costs, and shorter time to results (typically 30 min) compared to RT‐PCR (Reverse‐transcription Polymerase Chain Reaction). Building upon RT‐LAMP, digital LAMP (dLAMP) is a method that enables the absolute quantification of single nucleic acids in partitioned volumes, such as microfluidic wells or droplets.^14^, ^15^, ^16^, ^17^, ^18^ In droplet digital LAMP (ddLAMP), a sample is divided into thousands of picoliter‐sized drops, each serving as an individual reaction chamber.^18^, ^19^, ^20^ The compartmentalization provides several advantages, including increased sensitivity and reduced contamination.^14^, ^15^, ^16^, ^17^, ^18^, ^19^ Although it is known that the speed of RT‐LAMP amplification varies based on factors such as primers, template, and target concentrations,^21^ and prior work demonstrated that the speed of amplification of single molecules in a digital device correlated with multiple molecules in a PCR well plate,^22^ research into amplification speed as a function of droplet volume and chemistry remains relatively unexplored. Such investigations could facilitate the optimization of ddLAMP for rapid, point‐of‐care applications.

In this study, we introduce a digital droplet reverse transcription loop‐mediated isothermal amplification assay (ddRT‐LAMP) for the rapid detection of SARS‐CoV‐2 RNA. SARS‐CoV‐2 E gene templates were encapsulated with RT‐LAMP mix into microfluidic droplets and the time to detectable signal was studied as a function of drop size and DNA polymerase *Bst* 2.0 concentration. The drops were incubated at constant temperature of 64°C and drop fluorescence was monitored in real‐time. Amplification in drops was compared to bulk amplification using a standard qPCR machine. As expected, bulk assays exhibited longer assay times for lower target concentrations; yet surprisingly, we discover that ddRT‐LAMP significantly accelerated detection across a wide range of template concentrations. Notably, in drops, the lowest tested concentrations of 0.3 and 3 copies/μL were detected as rapidly as the highest concentration of 30 copies/μL within 12 min, compared to nearly an hour for the lowest concentrations in bulk. To explore the influence of droplet size on assay times, drops with diameters ranging from 30 to 400 μm were tested. Reducing droplet size did not consistently decrease assay times in smaller drops, as protein adsorption of the DNA polymerase *Bst* 2.0 at the higher oil‐water interfaces in smaller drops contributed to longer assay times compared to larger drops. This was tested by increasing polymerase concentration while holding drop size constant to compensate for the reduction in free *Bst* 2.0 polymerase available for the ddRT‐LAMP reaction. Optimization of both drop size and polymerase concentration yielded a quantitative ddRT‐LAMP method capable of detecting the SARS‐CoV‐2E gene with high sensitivity in as short as 8 min. Our results highlight the potential application of droplet encapsulation in reducing assay times, enabled by the rapid generation of fluorescent signal in small volumes. This development holds promise for the creation of rapid and sensitive point‐of‐care nucleic acid testing for the SARS‐CoV‐2 virus, as well as other pathogens.

## 2 RESULTS

### 2.1 A digital droplet RT‐LAMP assay (ddRT‐LAMP) for SARS‐CoV‐2 RNA detection

RT‐LAMP is a type of NAAT that undergoes an auto‐cycling strand displacement reaction at a constant temperature by using *Bst* polymerase, which greatly reduces the need for complex instrumentation and enables rapid nucleic acid strand amplification for detection within 30 min. Successful amplification is indicated by a high fluorescence signal from a DNA‐binding fluorescent dye, Syto 82, in the RT‐LAMP reaction mix, reflected by an increase in amplified products over time.

**FIGURE 1:**
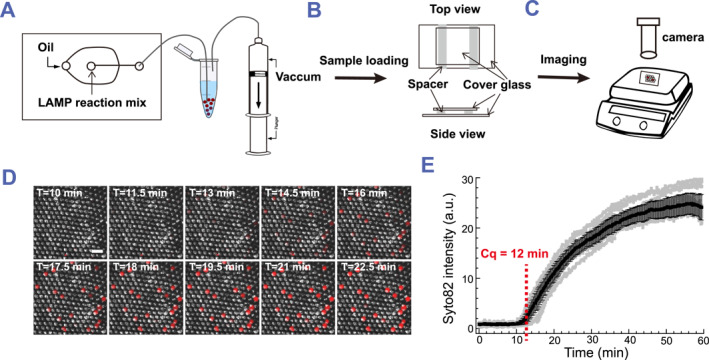
Droplet‐based real‐time LAMP assay. (A–C) Schematic of real‐time LAMP assay. (D) Images of merged bright field and Syto 82 channel for droplets with real‐time LAMP assay using Syto 82 as the indicator. Images are acquired 30 s per frame under camera on the hotplate. (E) Fluorescence intensity of Syto 82 in positive droplets over time. The gray curves represent Syto 82 intensity measured in each droplet. The black curve represents the averaged Syto 82 intensity. Data are shown as mean ± standard deviation.

To develop a digital droplet RT‐LAMP assay (ddRT‐LAMP) for SARS‐CoV‐2 detection, we used the SARS‐CoV‐2E gene as the nucleic acid template and performed RT‐LAMP reactions within microfluidic droplets. The RT‐LAMP reaction mix containing the SARS‐CoV‐2 E gene was introduced into a flow‐focusing microfluidic device with perpendicularly flowing oil to form droplets (Figure 1A). Using a simple syringe‐driven vacuum system,^23^ we encapsulated 25 μL RT‐LAMP reaction mix into 150 μm diameter droplets in under 1 min (Figure 1A). To perform and monitor RT‐LAMP reactions in droplets, we loaded the droplets into a glass chamber whose height is commensurate with the droplet diameter, ensuring a monolayer arrangement for improved heating and imaging (Figure 1B). We placed the glass chamber with the RT‐LAMP reaction mix on a pre‐heated hot plate set at 64°C and captured images at 30 s intervals for 1 h using a custom epifluorescence setup (Figure 1C).

Using ddRT‐LAMP to detect 30 copies/μL SARS‐CoV‐2 E gene, 5% of the total droplets yielded positive amplification results, and 98% of these positive droplets contained only a single copy of SARS‐CoV‐2 E gene based on the Poisson distribution.^24^ From the time‐lapse imaging data, we identified all positive droplets within the field of view (Figure 1D) and plotted the time dependence of the fluorescence intensity of the 20 fastest‐amplifying droplets (Figure 1E). We defined the “assay time” as the point when the second derivative of the intensity reaches its maximum, corresponding to the time when we detected exponential amplification of the E gene. The assay time for 30 copies/μL SARS‐CoV‐2E gene in 150 μm diameter droplets was 12 min.

### 2.2 Droplet encapsulation significantly enhances the detection speed of SARS‐CoV‐2 RNA compared to bulk

**FIGURE 2:**
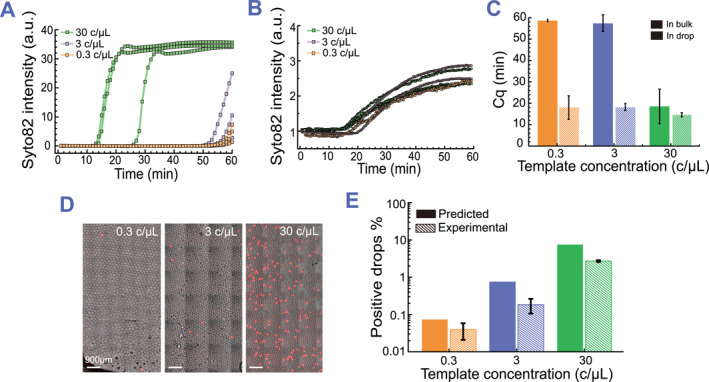
SARS‐CoV‐2 E gene amplification in bulk vs. in drop. (A) qRT‐LAMP assay in bulk. Representative amplification curves for template concentrations of 30 copies/μL, 3 copies/μL and 0.3 copies/μL are shown in green, purple and orange lines, respectively. (B) ddRT‐LAMP assay in 150 μm diameter droplets. Representative amplification curves for template concentrations of 30 copies/μL, 3 copies/μL and 0.3 copies/μL are shown in green, purple, and orange lines, respectively. (C) Assay time for ddRT‐LAMP reactions (gray filled bars) and qRT‐LAMP reactions (striped bars) with template concentrations of 0.3 copies/μL, 3 copies/μL and 30 copies/μL. Data are shown as mean ± standard deviation. (D) Images of merged brightfield and Syto 82 fluorescence channel showing ddRT‐LAMP reactions with template concentrations of 30 copies/μL, 3 copies/μL and 0.3 copies/μL. Images are taken after sample incubation at 64°C for 1 h. (E) Graph of actual positive drop percentage (open bars) and positive drop percentage predicted by Poisson loading calculation (gray bars).

For quantitative RT‐LAMP assays performed in bulk (qRT‐LAMP), nucleic acids with lower concentrations require more time to generate a detectable signal. To demonstrate this, we conducted triplicate qRT‐LAMP reactions for a serial dilution of SARS‐CoV‐2E gene at concentrations of 30 copies/μL, 3 copies/μL and 0.3 copies/μL in bulk (Figure 2A). These reactions were run in a qPCR machine set to a constant temperature of 64°C for 1 h. The corresponding average assay times were 18 min, 54 min, and 58 min, respectively, indicating that the detection speed depends on the initial SARS‐CoV‐2 E gene concentration. Our results are consistent with other qRT‐LAMP assays that use assay time as an indicator for quantifying nucleic acid concentration within a linear range.^25^, ^26^

To demonstrate that droplet encapsulation improves the detection speed of SARS‐CoV‐2 RNA, we generated 150 μm diameter droplet populations, each containing RT‐LAMP reaction mix with 30 copies/μL, 3 copies/μL and 0.3 copies/μL of the E gene, respectively. By continuously monitoring droplets using our custom epifluorescence setup for 1 h, we observed bright Syto 82 fluorescence in a fraction of droplets, indicating successful amplification of the SARS‐CoV‐2E gene. We analyzed the fluorescence intensity in positive droplets over time and found that ddRT‐LAMP assays for the E gene at different concentrations exhibited similar amplification curves (Figure 2B). In contrast to the delayed amplifications seen for 3 copies/μL and 0.3 copies/μL E gene in bulk, we discover that ddRT‐LAMP dramatically accelerated the detection of the amplification, allowing all concentrations to be detectable as quickly as the 30 copies/μL E gene condition (Figure 2C). This acceleration arises from the compartmentalization of E genes into picolitre droplets, which concentrates the amplified products and fluorescence signals. However, as the concentrations decrease, the number of positive droplets decreases as well. We analyzed approximately 1000 droplets from each population, and as expected, the number of positive droplets decreased by a factor of ∼10 for each subsequent decrease in concentration, from 30 copies/μL, to 3 copies/μL, and to 0.3 copies/μL (Figure 2D,E). Similar to other digital amplification methods, the number of positive droplets provides absolute quantification of nucleic acid concentration.

### 2.3 SARS‐CoV‐2 RNA detection by ddRT‐LAMP in different droplet sizes

**FIGURE 3:**
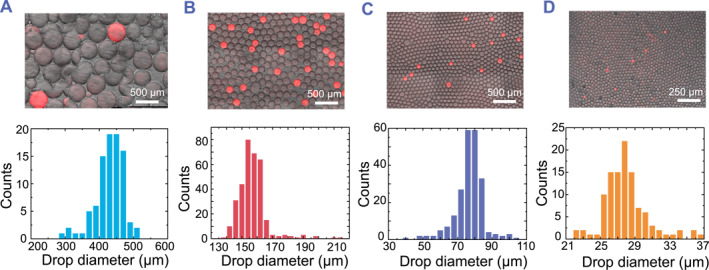
Effect of droplet size on ddRT‐LAMP reactions. (A–D) ddRT‐LAMP assay for 30 copies/μL E gene in (A) 400 μm diameter droplets, (B) 150 μm diameter droplets, (C) 80 μm diameter droplets, and (D) 30‐μm diameter droplets. Images were captured after incubating samples at 64°C for 1 h and shown as merged brightfield and Syto 82 fluorescence channels. Scale bars are 500 μm in (A–C) and 250 μm in (D).

As compartmentation concentrates the amplified products and fluorescence signals, we next investigated the effect of droplet size on the assay time to detection. We hypothesized that reducing the droplet size would decrease the time to detection due to high signal‐to‐noise ratio resulting from the rapid accumulation of fluorescence within these droplets confined volumes. Thus, we generated drops of different sizes, with diameters ranging from 30 to 400 μm, spanning a more than 2000‐fold difference in droplet volume (Figure 3A–D). The concentrations corresponding to a single copy of the E gene in 400 μm, 150 μm, 80 μm and 30 μm diameter droplets were 30 copies/μL, 600 copies/μL, 4000 copies/μL and 70,000 copies/μL, respectively (Supplementary Table S1).

**FIGURE 4:**
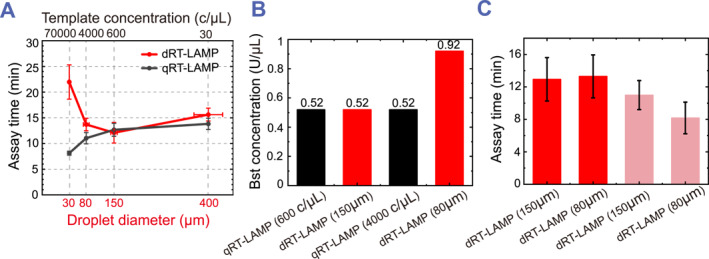
Effect of droplet size on ddRT‐LAMP reactions. (A) Assay time is plotted against template concentrations for qRT‐LAMP reactions (black curve, top *x*‐axis), and against droplet diameters for ddRT‐LAMP reactions with a template concentration of 30 copies/μL (red curve, bottom *x*‐axis). Template concentrations [E gene] in qRT‐LAMP reactions are correlated to droplet diameters (*d*) in ddRT‐LAMP reactions according to the relationship [E gene] = 6/(μ*d* ^3^). (B) Saturated *Bst* 2.0 concentrations in qRT‐LAMP reactions with template concentration of 600 copies/μL and 4000 copies/μL, and in ddRT‐LAMP reactions with droplet diameters of 150 and 80 μm. (C) Assay time for ddRT‐LAMP reactions with a droplet diameter of 150 versus 80 μm with *Bst* 2.0 saturation (red) and without *Bst* 2.0 saturation (pink). Data are shown as mean ± standard deviation.

To compare assay times in drops compared to bulk, we first performed bulk qRT‐LAMP with initial E gene concentrations equivalent to those in different droplet sizes (Figure 4A and Supplementary Table S1). The assay time for qRT‐LAMP decreases from 15 to 8 min when the initial E gene concentration increases from 30 copies/μL to 70,000 copies/μL, indicating that the assay time for ddRT‐LAMP in smaller droplets should also exhibit a comparable reduction. To test the hypothesis that smaller droplet diameters decrease assay time, we performed ddRT‐LAMP assay in droplets with diameters of 30 μm, 80 μm, 150 μm, and 400 μm. Indeed, the assay time decreased from ∼16 to ∼12 min when the droplet diameter decreases from 400 to 150 μm (Figure 4A, red curve, and Supplementary Figure S1). However, when the droplet diameter decreased to 80 and 30 μm, the assay time increased to 14 and 22 min, respectively (Figure 4A, red curve, and Supplementary Figure S1). This unexpected deviation suggested that other factors must increase the assay time as the droplet size decreases.

### 2.4 Protein adsorption at the oil–water interface delays SARS‐CoV‐2 RNA detection

Previous studies have demonstrated that proteins can adsorb to oil–water interfaces due to the presence of both hydrophobic and hydrophilic domains.^27^, ^28^, ^29^ We hypothesized that in our ddRT‐LAMP assay, the unexpected slowdown in assay time in smaller droplets resulted from surface adsorption at the oil‐water interface, and specifically from adsorption of the *Bst* 2.0 polymerase in the RT‐LAMP reaction. We hypothesized this protein adsorption at the oil‐water interfaces in smaller droplets would lead to a reduction in the concentration of free *Bst* 2.0 available for the ddRT‐LAMP reaction, resulting in longer assay times in smaller droplets.

To test this hypothesis, we compared ddRT‐LAMP assay times in 80 and 150 μm diameter droplets after adding additional *Bst* 2.0 to the reaction mix in the droplets. For the 150 μm diameter droplets, we observed that the assay time decreased from ∼13 to ∼11 min as the *Bst* 2.0 increased concentration from 0.32 U/μL to 0.52 U/μL. Further increases in the *Bst* 2.0 concentration maintained the assay time at ∼11 min, indicating that the *Bst* 2.0 for ddRT‐LAMP reaction in 150 μm diameter droplets became saturated at 0.52 U/μL (Supplementary Figures S2A and S4B). This saturated *Bst* 2.0 concentration for the ddRT‐LAMP reaction in 150 μm diameter droplets aligned with that in the qRT‐LAMP reaction (template concentration: 600 copies/μL) (Figure 4B), suggesting that less *Bst* 2.0 is adsorbed at the water‐oil interface of 150 μm diameter droplets.

However, unlike the qRT‐LAMP reaction (template concentration: 4000 copies/μL), where the *Bst* 2.0 concentration saturated at 0.52 U/μL with an assay time of only 8 min, the assay time for the ddRT‐LAMP reaction in 80 μm diameter droplets did not saturate at a *Bst* 2.0 concentration of 0.52 U/μL. Instead, it continued to decrease as more *Bst* 2.0 was added. This result indicates that overcoming higher surface areas by saturating the ddRT‐LAMP in smaller droplets requires a higher concentration of *Bst* 2.0 (Supplementary Figure S2B). At the highest *Bst* 2.0 concentration tested (0.92 U/μL), we found that the assay time for ddRT‐LAMP in 80 μm diameter droplets indeed decreased to 8 min, consistent with the assay time for the corresponding qRT‐LAMP reaction (Supplementary Figure S2B). In summary, our results demonstrate that the addition of extra *Bst* 2.0 can compensate for protein adsorption at the oil‐water interface of smaller droplets, resulting in shorter assay times for ddRT‐LAMP in 80 μm diameter droplets compared to 150 μm diameter droplets (Figure 4C).

## 3 CONCLUSIONS

Our study demonstrates that the detection speed of SARS‐CoV‐2 RNA can be significantly reduced by compartmentalizing RT‐LAMP assays into droplets. Compared to bulk RT‐LAMP assays, compartmentalization greatly enhances detection speed by concentrating the amplified nucleic acids and resulting fluorescence signals. As ddRT‐LAMP is a fundamentally digital technique in which templates either exhibit post‐amplification fluorescence in the partitioned drops or do not, the assay time remains independent of the initial nucleic acid concentration. This was evidenced by our experiments demonstrating that regardless of template concentration, the lowest tested concentrations of 0.3 and 3 copies/μL were detectable as quickly as the highest tested concentration of 30 copies/μL in 12 min. This is in contrast to the RT‐LAMP assays performed in bulk, where the two lower concentrations required nearly an hour to generate a detectable signal. Thus, ddRT‐LAMP is not dependent on new biochemistry but rather on concentrating the fluorescence signal in droplets. This enables rapid detection of low concentrations of viral RNA template due to its high sensitivity and signal‐to‐noise when compared to bulk qRT‐LAMP.

Our findings also demonstrate that increasing concentration of the DNA polymerase to compensate for interfacial adsorption can further accelerate the detection speed. By reducing droplet size and adjusting droplet chemistry, we can detect the SARS‐CoV‐2 E gene within just 8 min using the ddRT‐LAMP assay in 80 μm diameter droplets.

Compartmentalization has been shown to be compatible with other NAATs, such as polymerase chain reaction (PCR),^19^, ^30^, ^31^ strand displacement assay (SDA),^32^ and CRISPR.^32^ Future research focused on understanding the effect of varying droplet sizes and surface adsorption conditions on assay times for other NAATs would lead to optimization of drop‐based digital amplification methods. Such studies could offer valuable insights into the general behavior of these assays when performed within droplets.

Overall, we have demonstrated that ddRT‐LAMP is suitable for rapid and quantitative testing of viral RNA. In addition to the advantage of increased assay speeds in drops, this method shows promise for the development of sensitive point‐of‐care nucleic acid testing, due to the minimization of specialized equipment and relatively low cost of RT‐LAMP compared to other NAATs. We anticipate that the results of this work will contribute to the high‐speed and quantitative detection of SARS‐CoV‐2, as well as other pathogens, and will advance our understanding of the effects of droplet size and interfacial surface area on assay speeds.

## 4 MATERIALS AND METHODS

### 4.1 Template preparation

Aliquots of SARS‐CoV‐2 E gene at 10^8^ copies/μL were diluted to the desired concentrations with nuclease‐free water (NEB B1500L). The aliquots of the E gene are kept at −80°C until use. The sequence for E gene is shown as follows (5′ to 3′): ATGTTACCTTCTTCATCTACAATAAAATTGTTGATGAGCCTGAAGAACATGTCCAAATTGTACCAATCGACGGTTCATCCGGAGTTGTTAATCCAGTAATGGAAGATTCGCACGGTCCAATTTATGATGAACCGACGACGACTACTAGCGTGCCTTTGTAAGCACAAGCTGATGAGTACGAACTTATGTACTCATTCGTTTCGGAAGAGACAGGTACGTTAATAGTTAATAGCGTACTTCTTTTTCTTGCTTTCGTGGTATTCTTGCTAGTTACACTAGCCATCCTTACTGCGCTTCGATTGTGTGCGTACTGCTGCAATATTGTTAACGTGAGTCTTGTAAAACCTTCTTTTTACGTTTACTCTCGTGTTAAAAATCTGAATTCTTCTAGAGTTCCTGATCTTCTGGTCTAAACGAACTAAATATTATATTAGTTTTTCTGTTTGGAACTTTAATTTTAGCCATGGCAGATTCCAA.

### 4.2 RT‐LAMP primers for SARS‐CoV‐3 E gene template

All primers for the E gene were ordered from Integrated DNA Technologies. We resuspend all primers to 50 μM in nuclease‐free water (NEB B1500L). The SARS‐CoV‐2 primer mix for E gene was prepared as a 10X stock solution in the following manner: 80 μL of FIP, 80 μL of BIP, 20 μL of LF, 20 μL of LB, 10 μL of F3, 10 μL of B3, and 30 μL nuclease‐free water (250 μL total volume). The primer stocks and the 10X primer stocking solution were stored at −20°C until use. The sequences of all E gene RT‐LAMP primers are listed as follows^33^:

**Table:** 

Name	Sequence (5′ to 3′)
FIP	ACCACGAAAGCAAGAAAAAGAAGTTCGTTTCGGAAGAGACAG
BIP	TTGCTAGTTACACTAGCCATCCTTAGGTTTTACAAGACTCACGT
LF	CGCTATTAACTATTAACG
LB	GCGCTTCGATTGTGTGCGT
F3	TGAGTACGAACTTATGTACTCAT
B3	TTCAGATTTTTAACACGAGAGT

### 4.3 RT‐LAMP reaction mix

We prepare a 25 μL RT‐LAMP reaction mix according to the following: 12.5 μL Warm Start 2X Master Mix (NEB M1700S), 0.175 μL 100 mM dUTP (NEB N0459S), 0.5 μL 1000 U/mL Antarctic Thermolabile UDG (NEB M0372L), 2.5 μL 2 M Guanidine HCl (Promega H5381), 2.325 μL of 5 mM TCEP (EMD Millipore 51805), 0.5 μL of 25 μM Syto 82 (Invitrogen S11363), 2 μL Nuclease‐free water, 2.5 μL 10X primer mix, and 2 μL E gene template. The qRT‐LAMP reaction was performed on a qPCR machine (Bio‐Rad, CFX96). The parameters were set for isothermal amplification as follows: 1: 65°C for 30 s, 2: 65°C for 1 min and Plate Read, 3: GOTO 2, 59 more times, 4: 4°C, *∞*.

### 4.4 Microfluidic chip design and fabrication

Microfluidic chips were fabricated from polydimethylsiloxane (PDMS) polymer (Dow Corning, Sylgard 184) according to published methods.^34^ SU8‐3000 series photoresist (Microchem) was used to fabricate the master templates. After the PDMS slab was cut from the mold, we punched inlet and outlet holes using a biopsy punch (1 mm diameter; Ted Pella, Harris Uni‐Core, cat. no. 15110‐10). The hole‐punch debris was removed using transparent adhesive tape. The PDMS was bonded to a glass microscope slide to form the final microfluidic device. To make the PDMS chip hydrophobic for generating aqueous droplets, the chip channels were treated with Aquapel (Aquapel, cat. no. 47100) for 30 min and the device was dried in a 65°C oven before use.

### 4.5 Droplet generation

We encapsulated an aqueous reaction mix consisting of the RT‐LAMP reagents, template, and associated primers in a monodisperse emulsion with HFE7500 fluorocarbon oil (Novec™, 3M) containing 2% (w/v) Perfluoropolyether‐polyethylene glycol‐perfluoropolyether triblock surfactant^23^ (008‐FluoroSurfactant, Ran Biotechnologies) as the continuous phase. This was accomplished by loading the reaction mix and the fluorinated oil into a flow‐focusing microfluidic dropmaking device. We used pipette tips as reservoirs to hold the reaction mix at the aqueous inlet of our devices and a 60‐mL syringe to generate vacuum at the outlet of the device. The pressure difference resulted in flow through the device, yielding a monodisperse emulsion whose characteristic size was set by the dropmaking geometry and the applied vacuum pressure.

### 4.6 Chamber making and droplet loading

After generation, droplets were loaded into a custom glass chamber. The chamber was created using two cover glasses separated by a spacer of known thickness. The cover glass with size 50 mm × 22 mm (VWR, Cat# 16004‐314) was used as the bottom of the chamber, while the one with size 18 mm × 18 mm (VWR, Cat# 48366045) served as the top. Spacers (McMaster‐Carr, Cat# 9513K42) with thicknesses of 300 μm, 150 μm, 80 μm and 30 μm were sandwiched between the two cover glasses to create chambers with heights of 300 μm, 150 μm, 80 μm and 30 μm, respectively. Prior to loading the chamber, a UV epoxy, Norland 81 (Norland Products, Cat# NOA81), was used to glue the two cover glasses and the spacer together. After applying the Norland 81, the assembly was treated under UV light (365 nm) for 1 h to cure the UV epoxy before being loaded with droplets. After loading, we applied a quick‐curing epoxy (Devcon, #14250) on the four sides of the chamber to seal it.

### 4.7 Droplet incubation and imaging

We placed the glass chamber with droplets on a pre‐heated hot plate, which was set at 64°C. We imaged the sample on the hot plate with a custom fluorescence microscope. The Syto 82 dye was excited with a 530 nm LED (Thorlabs, Cat# M530L4) and imaged through a 550 LP filter (Thorlabs, Cat# FELH0550). After placing the sample on the hot plate, we imaged the sample every 30 s for 1 h in total.

### 4.8 Software, image processing and statistical analysis

AutoCad (AutoDesk) software was used to design the mask for lithography. Image processing and analysis were performed using ImageJ^35^ (National Institutes of Health). Origin (OriginLab) was used for plotting graphs. Each experiment was performed independently at minimum in duplicate.

## AUTHOR CONTRIBUTIONS

Yuan Yuan: investigation (lead), conceptualization, writing original draft and review & editing. Perry Ellis: investigation (supporting), conceptualization, supervision, writing original draft and review & editing. Ye Tao, Dimitri A. Bikos, Emma K. Loveday, Mallory M. Thomas, James N. Wilking and Connie B. Chang: investigation (supporting), writing ‐ review & editing. Fangfu Ye: funding acquisition, supervision, writing ‐ review & editing. David A. Weitz: conceptualization, funding acquisition, supervision, writing original draft and review & editing.

## CONFLICT OF INTEREST STATEMENT

David A. Weitz is an editor‐in‐chief, and Fangfu Ye an associate editor of *Smart Medicine*. They were not involved in the editorial review or the decision to publish this article. All authors declare that there are no competing interests.

## ETHICS STATEMENT

There are no experiments dealing with animal, human subjects or tissue samples from human subjects in this study.

## Supporting information

Supporting Information S1

## References

[1] H. Wang , G. Li , J. Zhao , Y. Li , Y. Ai , Front. Med. 2021, 7, 571709. 10.3389/fmed.2020.571709 PMC7848129 33537322

[2] V. M. Corman , O. Landt , M. Kaiser , R. Molenkamp , A. Meijer , D. K. W. Chu , T. Bleicker , S. Brünink , J. Schneider , M. L. Schmidt , D. G. J. C. Mulders , B. L. Haagmans , B. van der Veer , S. van den Brink , L. Wijsman , G. Goderski , J. L. Romette , J. Ellis , M. Zambon , M. Peiris , H. Goossens , C. Reusken , M. P. G. Koopmans , C. Drosten , Euro Surveill. 2020, 25, 2000045.

[3] J. P. Broughton , X. Deng , G. Yu , C. L. Fasching , V. Servellita , J. Singh , X. Miao , J. A. Streithorst , A. Granados , A. Sotomayor‐Gonzalez , K. Zorn , A. Gopez , E. Hsu , W. Gu , S. Miller , C. Y. Pan , H. Guevara , D. A. Wadford , J. S. Chen , C. Y. Chiu , Nat. Biotechnol. 2020, 38, 870. 32300245 10.1038/s41587-020-0513-4 PMC9107629

[4] L. Yu , S. Wu , X. Hao , X. Dong , L. Mao , V. Pelechano , W. H. Chen , X. Yin , Clin. Chem. 2020, 66, 975. 32315390 10.1093/clinchem/hvaa102 PMC7188121

[5] V. T. Chu , N. G. Schwartz , M. A. P. Donnelly , M. R. Chuey , R. Soto , A. R. Yousaf , E. N. Schmitt‐Matzen , S. Sleweon , J. Ruffin , N. Thornburg , J. L. Harcourt , A. Tamin , G. Kim , J. M. Folster , L. J. Hughes , S. Tong , G. Stringer , B. A. Albanese , S. E. Totten , M. M. Hudziec , S. R. Matzinger , E. A. Dietrich , S. W. Sheldon , S. Stous , E. C. McDonald , B. Austin , M. E. Beatty , J. E. Staples , M. E. Killerby , C. H. Hsu , J. E. Tate , H. L. Kirking , A. Matanock , COVID‐19 Household Transmission Team , JAMA Intern. Med. 2022, 182, 701. 35486394 10.1001/jamainternmed.2022.1827 PMC9055515

[6] M. Wölfl‐Duchek , F. Bergmann , A. Jorda , M. Weber , M. Müller , T. Seitz , A. Zoufaly , R. Strassl , M. Zeitlinger , H. Herkner , H. Schnidar , K. Anderle , U. Derhaschnig , Microbiol. Spectrum 2022, 10, e02029‐21. 10.1128/spectrum.02029-21 PMC8809344 35107327

[7] P. Kralik , M. Ricchi , Front. Microbiol. 2017, 8, 108. 10.3389/fmicb.2017.00108 PMC5288344 28210243

[8] Q. Yang , T. K. Saldi , P. K. Gonzales , E. Lasda , C. J. Decker , K. L. Tat , M. R. Fink , C. R. Hager , J. C. Davis , C. D. Ozeroff , D. Muhlrad , S. K. Clark , W. T. Fattor , N. R. Meyerson , C. L. Paige , A. R. Gilchrist , A. Barbachano‐Guerrero , E. R. Worden‐Sapper , S. S. Wu , G. R. Brisson , M. B. McQueen , R. D. Dowell , L. Leinwand , R. Parker , S. L. Sawyer , Proc. Natl. Acad. Sci. U. S. A. 2021, 118, e2104547118. 10.1073/pnas.2104547118 PMC8166196 33972412

[9] B. A. Rabe , C. Cepko , Proc. Natl. Acad. Sci. U. S. A. 2020, 117, 24450. 32900935 10.1073/pnas.2011221117 PMC7533677

[10] M. Inaba , Y. Higashimoto , Y. Toyama , T. Horiguchi , M. Hibino , M. Iwata , K. Imaizumi , Y. Doi , Int. J. Infect. Dis. 2021, 107, 195. 33862213 10.1016/j.ijid.2021.04.018 PMC8056478

[11] J. García‐Bernalt Diego , P. Fernández‐Soto , M. Domínguez‐Gil , M. Belhassen‐García , J. L. M. Bellido , A. Muro , Diagnostics 2021, 11, 438. 33806456 10.3390/diagnostics11030438 PMC8000859

[12] V. L. Dao Thi , K. Herbst , K. Boerner , M. Meurer , L. P. M. Kremer , D. Kirrmaier , A. Freistaedter , D. Papagiannidis , C. Galmozzi , M. L. Stanifer , S. Boulant , S. Klein , P. Chlanda , D. Khalid , I. B. Miranda , P. Schnitzler , H. G. Kräusslich , M. Knop , S. Anders , Sci. Transl. Med. 2020, 12, eabc7075. 10.1126/scitranslmed.abc7075 PMC7574920 32719001

[13] X. Huang , G. Tang , N. Ismail , X. Wang , EBioMedicine 2022, 75, 103736. 10.1016/j.ebiom.2021.103736 PMC8674011 34922321

[14] H. Yuan , Y. Chao , H. C. Shum , Small 2020, 16, 1904469. 10.1002/smll.201904469 31899592

[15] A. Gansen , A. M. Herrick , I. K. Dimov , L. P. Lee , D. T. Chiu , Lab Chip 2012, 12, 2247. 22399016 10.1039/c2lc21247a PMC3383853

[16] Q. Zhu , Y. Gao , B. Yu , H. Ren , L. Qiu , S. Han , W. Jin , Q. Jin , Y. Mu , Lab Chip 2012, 12, 4755. 22986619 10.1039/c2lc40774d

[17] B. Sun , F. Shen , S. E. McCalla , J. E. Kreutz , M. A. Karymov , R. F. Ismagilov , Anal. Chem. 2013, 85, 1540. 23324061 10.1021/ac3037206 PMC3578705

[18] F. Schuler , C. Siber , S. Hin , S. Wadle , N. Paust , R. Zengerle , F. von Stetten , Anal. Methods 2016, 8, 2750.

[19] H. N. Vasudevan , P. Xu , V. Servellita , S. Miller , L. Liu , A. Gopez , C. Y. Chiu , A. R. Abate , Sci. Rep. 2021, 11, 780. 33436939 10.1038/s41598-020-80715-1 PMC7804156

[20] T. D. Rane , L. Chen , H. C. Zec , T. H. Wang , Lab Chip 2015, 15, 776. 25431886 10.1039/c4lc01158a PMC4626017

[21] N. A. Tanner , Y. Zhang , T. C. Evans , BioTechniques 2012, 53, 81. 23030060 10.2144/0000113902

[22] E. M. Khorosheva , M. A. Karymov , D. A. Selck , R. F. Ismagilov , Nucleic Acids Res. 2016, 44, e10. 26358811 10.1093/nar/gkv877 PMC4737171

[23] A. R. Abate , D. A. Weitz , Biomicrofluidics 2011, 5, 014107. 10.1063/1.3567093 PMC3073010 21483661

[24] A. S. Basu , SLAS Technol. 2017, 22, 369. 28448765 10.1177/2472630317705680

[25] D. He , J. Yang , X. Jiang , Y. Lin , H. Chen , Y. Tang , Y. Diao , Poult. Sci. 2020, 99, 6586. 33248574 10.1016/j.psj.2020.09.077 PMC7705033

[26] G. Papadakis , A. K. Pantazis , N. Fikas , S. Chatziioannidou , V. Tsiakalou , K. Michaelidou , V. Pogka , M. Megariti , M. Vardaki , K. Giarentis , J. Heaney , E. Nastouli , T. Karamitros , A. Mentis , A. Zafiropoulos , G. Sourvinos , S. Agelaki , E. Gizeli , Sci. Rep. 2022, 12, 3775. 35260588 10.1038/s41598-022-06632-7 PMC8904468

[27] F. Su , G. Li , Y. Fan , Y. Yan , Sci. Rep. 2016, 6, 29670. 27424490 10.1038/srep29670 PMC4947930

[28] S. Rusli , J. Grabowski , A. Drews , M. Kraume , Processes 2020, 8, 1082.

[29] M. Campana , S. L. Hosking , J. T. Petkov , I. M. Tucker , J. R. P. Webster , A. Zarbakhsh , J. R. Lu , Langmuir 2015, 31, 5614. 25875917 10.1021/acs.langmuir.5b00646

[30] C. Alteri , V. Cento , M. Antonello , L. Colagrossi , M. Merli , N. Ughi , S. Renica , E. Matarazzo , F. Di Ruscio , L. Tartaglione , J. Colombo , C. Grimaldi , S. Carta , A. Nava , V. Costabile , C. Baiguera , D. Campisi , D. Fanti , C. Vismara , R. Fumagalli , F. Scaglione , O. M. Epis , M. Puoti , C. F. Perno , PLoS One 2020, 15, e0236311. 32898153 10.1371/journal.pone.0236311 PMC7478621

[31] S. C. Taylor , G. Laperriere , H. Germain , Sci. Rep. 2017, 7, 2409. 28546538 10.1038/s41598-017-02217-x PMC5445070

[32] M. C. Giuffrida , L. M. Zanoli , R. D’Agata , A. Finotti , R. Gambari , G. Spoto , Anal. Bioanal. Chem. 2015, 407, 1533. 25579461 10.1007/s00216-014-8405-4

[33] Y. Zhang , G. Ren , J. Buss , A. J. Barry , G. C. Patton , N. A. Tanner , BioTechniques 2020, 69, 179. 10.2144/btn-2020-0078 32635743

[34] Y. Yuan , J. Brouchon , J. M. Calvo‐Calle , J. Xia , L. Sun , X. Zhang , K. L. Clayton , F. Ye , D. A. Weitz , J. A. Heyman , Lab Chip 2020, 20, 1513. 32242586 10.1039/c9lc01261c PMC7313394

[35] C. T. Rueden , J. Schindelin , M. C. Hiner , B. E. DeZonia , A. E. Walter , E. T. Arena , K. W. Eliceiri , BMC Bioinf. 2017, 18, 529. 10.1186/s12859-017-1934-z PMC5708080 29187165

