# Supplementary material for: Digital droplet RT‐LAMP increases speed of SARS‐CoV‐2 viral RNA detection
Source: Smart Med. 2024 Jun 5;3(2):e20240008. doi: 10.1002/SMMD.20240008 (PMC11235653; doi:10.1002/SMMD.20240008)
Supplement: Supplementary file 1 — Supporting Information S1 [file SMMD-3-e20240008-s001.docx]

**Supplementary Information for**

**Digital droplet RT-LAMP increases speed of SARS-CoV-2 viral RNA detection**

Yuan Yuan^1,2†^, Perry Ellis^2†^, Ye Tao^2^, Dimitri A. Bikos^3,4^, Emma K. Loveday^3,4^, Mallory M. Thomas^3,4^,

James N. Wilking^3,4,8^, Connie B. Chang^3,4,8^, Fangfu Ye^1,5*^ and David A. Weitz^2,6,7*^

**Affiliations**

^1^ Oujiang Laboratory (Zhejiang Lab for Regenerative Medicine, Vision and Brain Health); Wenzhou Institute, University of Chinese Academy of Sciences, Wenzhou, Zhejiang 325000, China

^2^ John A. Paulson School of Engineering and Applied Sciences, Harvard University, Cambridge, MA 02138, USA

^3^ Department of Chemical and Biological Engineering, Montana State University, Bozeman, MT 59717, USA

^4^ Center for Biofilm Engineering, Montana State University, Bozeman, MT 59717, USA

^5^ Beijing National Laboratory for Condensed Matter Physics, Institute of Physics, Chinese Academy of Sciences, Beijing 100190, China

^6^ Department of Physics, Harvard University, Cambridge, MA 02138, USA

^7^ Wyss Institute for Biologically Inspired Engineering, Harvard University, Boston, MA 02115, USA

^8^ Department of Physiology and Biomedical Engineering, Mayo Clinic, Rochester, MN 55905

^†^ These authors contributed equally.

* Corresponding authors: David A. Weitz ([weitz@seas.harvard.edu](mailto:weitz@seas.harvard.edu)); Fangfu Ye ([fye@iphy.ac.cn](mailto:fye@iphy.ac.cn))


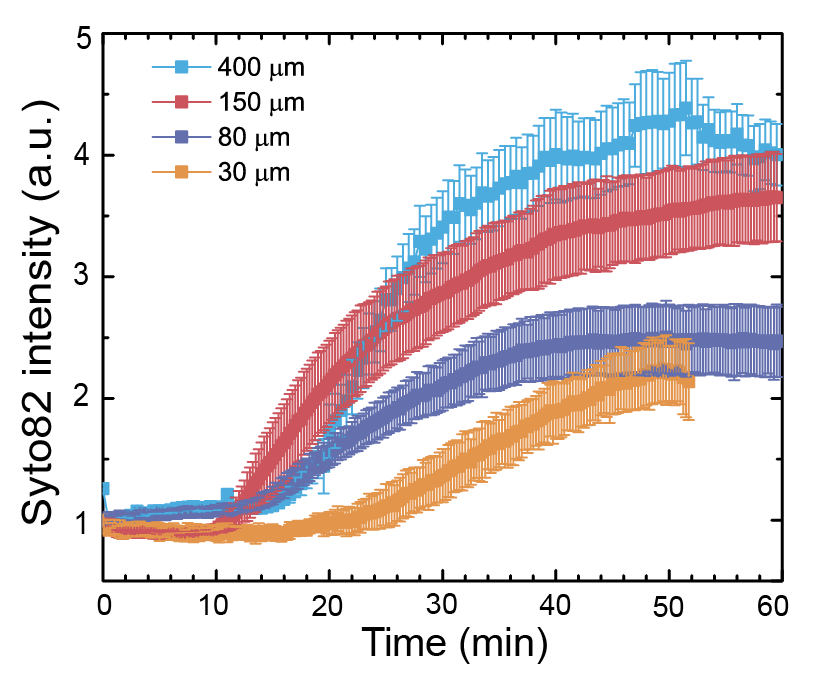


**Supplementary Figure 1.** Fluorescence intensity of Syto 82 in positive droplets with different droplet sizes over time. Data are shown as mean ± standard deviation.

**
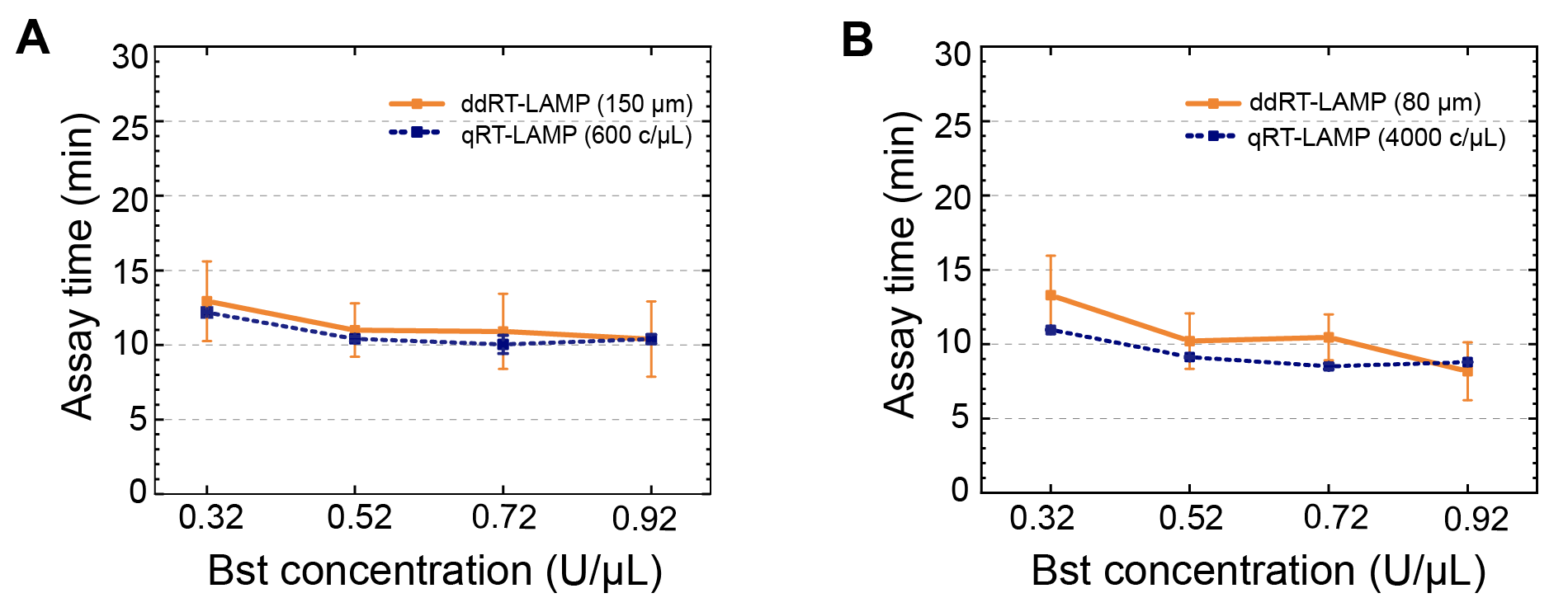
Supplementary Figure 2. Assay time of ddRT-LAMP and qRT-LAMP as a function of *Bst* 2.0 concentration. A)** Assay time of ddRT-LAMP reactions in 150 μm diameter droplets (orange line and squares) versus that of qRT-LAMP reactions with 600 copies/μL template (dashed blue line and squares) with different *Bst* 2.0 concentrations. **B)** Assay time of ddRT-LAMP reactions in 80 μm diameter droplets (dashed blue line and squares) versus that of qRT-LAMP reactions with 4000 copies/μL template (dashed blue line and squares) with different *Bst* 2.0 concentrations. Data are shown as mean ± standard deviation.

**Supplementary Table 1.** Single template concentrations in drops equivalent to bulk template concentrations in copies/μL.

| **One template in drop** | **In bulk** |
| --- | --- |
| 400 μm | 30 copies/μL |
| 150 μm | 600 copies/μL |
| 80 μm | 4000 copies/μL |
| 30 μm | 70000 copies/μL |
